# Molecular characterization of ring chromosome 18 by low-coverage next generation sequencing

**DOI:** 10.1186/s12881-015-0206-x

**Published:** 2015-07-30

**Authors:** Xiuqing Ji, Dong Liang, Ruihong Sun, Cuiyun Liu, Dingyuan Ma, Yan Wang, Ping Hu, Zhengfeng Xu

**Affiliations:** State Key Laboratory of Reproductive Medicine, Department of Prenatal Diagnosis, Nanjing Maternity and Child Health Care Hospital Affiliated to Nanjing Medical University, Nanjing, China; Department of Laboratory Medicine, The First Affiliated Hospital of Nanjing Medical University, Nanjing, China; National Key Clinical Department of Laboratory Medicine, Nanjing, China

**Keywords:** Ring chromosome, Breakpoint, Next generation sequencing

## Abstract

**Background:**

Ring chromosomes are one category of structurally abnormal chromosomes that can lead to severe growth retardation and other clinical defects. Traditionally, their diagnosis and characterization has largely relied on conventional cytogenetics and fluorescence in situ hybridization, array-based comparative genomic hybridization and single nucleotide polymorphism array-based comparative genomic hybridization. However, these methods are ineffectively at characterizing the ring chromosome structure and only offer a low resolution mapping of breakpoints. Here, we applied whole-genome low-coverage paired-end next generation sequencing (NGS) to two suspected cases of ring chromosome 18 (r(18)) and characterized the ring structure including the chromosome dosage changes and the breakpoint junction.

**Methods:**

The breakpoints and chromosome copy number variations (CNVs) of r(18) were characterized by whole-genome low-coverage paired-end NGS. We confirmed the dosage change by single nucleotide polymorphisms array, and validated the junction site regions using PCR followed by Sanger sequencing.

**Results:**

We successfully and fully characterized the r(18) in two cases by NGS. We mapped the breakpoints with a high resolution and identified all CNVs in both cases. We analyzed the breakpoint regions and discovered two breakpoints located within repetitive sequence regions, and two near the repetitive sequence regions. One of the breakpoints in case 2 was located within the gene *METTL4,* while the other breakpoints were intergenic.

**Conclusions:**

We demonstrated that whole-genome low-coverage paired-end NGS can be used directly to map breakpoints with a high molecular resolution and detect all CNVs on r(18). This approach will provide new insights into the genotype-phenotype correlations on r(18) and the underlying mechanism of ring chromosomes formation. Our results also demonstrate that this can be a powerful approach for the diagnosis and characterization of ring chromosomes in the clinic.

**Electronic supplementary material:**

The online version of this article (doi:10.1186/s12881-015-0206-x) contains supplementary material, which is available to authorized users.

## Background

Ring chromosomes are a structurally abnormal type of chromosome, which usually arise following breakages in the short and long arms of chromosomes and fusions at the breakpoints. They are often accompanied by loss of distal chromosome segments. The phenomenon can occur on any human chromosomes, although chromosomes 13 and 18 are the most commonly affected [1]. Ring chromosome 18 (r(18)), deletion 18p (18p-) and deletion 18q (18q-) have an overall incidence of approximately 1 in 40,000 live human births [2].

Notable technological advances have been made in the identification of ring chromosomes. Low-resolution conventional cytogenetics was used initially [3], then molecular cytogenetic approaches combining fluorescence in situ hybridization (FISH) together with polymerase chain reaction (PCR) were applied to map the breakpoints in r(18) [4]. More recently, array comparative genomic hybridization (aCGH) [5] and single-nucleotide polymorphism array (SNP-array) [6] have enabled the more precise evaluation of breakpoints, with a resolution of up to 0.1 Mb.

Next-generation paired-end sequencing (NGS), which yields millions of paired short reads from the ends of fragments of predetermined size, has also been applied to the genome-wide detection of chromosome structural variations [7]. Recent studies using different DNA preparation protocols and sequencing platform demonstrated that NGS was able to characterize chromosome translocations and inversions with a high resolution, and with a base genome coverage as low as 1X [8–13]. However, ring chromosomes, which always involve chromosome dose changes and structural rearrangements, have not yet been characterized by NGS.

Here, we implemented a whole-genome low-coverage paired-end NGS method with the aim of capturing all breakpoints at a high resolution and identifying all copy number variations (CNVs) in a single experiment to fully describe the molecular characterizations of r(18). We applied this approach to two suspected cases of r(18), and completely characterized the chromosome breakpoints of these cases at a base pair level.

## Methods

### Subjects

The parents of the case 1 fetus were a 31-year-old woman and a 32-years-old man. Both were in good health, had no abnormal family history, and had not been exposed to teratogenic agents before or during the pregnancy. The fetus was found to be affected by nuchal cystic hygromas at 18 weeks of gestation through an obstetric ultrasound examination, and then ventricular septal defects (VSD), a single umbilical artery, and nuchal cystic hygromas (35.6 × 20.4 mm in size with two cavities) at 22 weeks by a level II ultrasound examination. Amniocentesis was undertaken at the 19th week, and routine G-bands by trypsin using Giemsa (GTG) analysis indicated an abnormal female karyotype: 46,XX, r(18)[27]/45,XX,-18[5]?. After genetic consulation, the parents opted for termination of the pregnancy at 22 weeks’ gestation. An autopsy revealed nuchal cystic hygromas, VSD, low-set ears, but no other internal or external malformations. GTG analysis was also performed for the parents, who were both revealed to have a normal karyotype.

The 8-month-old case 2 patient was the first child of healthy, non-consanguineous parents: a 29-year-old mother, and a 30-year-old father. No abnormal family history was reported, and there had been no exposure to teratogenic agents before or during the pregnancy. The pregnancy was uneventful, with normal ultrasound and serum examinations reports. On examination at the age of 8 months, the female patient displayed microcephaly, developmental retardation, orbital hypertelorism and ptosis of the upper eyelid. Brain magnetic resonance imaging revealed no structural abnormalities, and no abnormalities were found in the internal organs. The karyotype of 46, XX, r(18)? was detected by GTG analysis in the peripheral blood. Both parents had a normal karyotype.

This study was approved by the Medicine Ethics Committee of Nanjing Maternity and Child Health Care Hospital. The parents of both patients signed an informed consent form in our study.

### Cytogenetic analysis and single nucleotide polymorphism (SNP) array

For cytogenetic analysis, GTG banding at the 400 to 550-band level was performed on both cases according to a standard protocol.

Genomic DNA was extracted from peripheral blood or amniotic fluid cell from patients and controls using the QIAamp DNA Mini Kit (Qiagen, Hilden, Germany). The human cyto12 SNP-array (Illumina, San Diego, CA) comprising around 300,000 SNPs was applied for the whole genome scan in both cases. SNP array experiments were carried out as previously described [14]. Molecular karyotype analysis was performed by KaryoStudio V 1.3.11 (Illumina). The evaluation of CNVs pathogenicity was based on the gene content according to human assembly hg19/GRCh37.1 (hereafter referred to hg19).

### Whole-genome low-coverage paired-end NGS

DNA samples from both patients were tested using a whole-genome low-coverage paired-end NGS as described previously. [12]. Briefly, 3 μg of genomic DNA was sheared using HydroShear device (GeneMachine, San Carlos, CA) to construct a library with insert size of 3–8 kb. DNA fragments were end-repaired and 3'-end labeled with biotinylated nucleotides. After circularization via intramolecular ligation, they were then sheared again using the Covaris S2 sonicator to generate fragments of ~500 bp. These fragments were then purified using streptavidin-coated magnetic beads, end-repaired and A-tailed in preparation for ligation to Illumina paired-end oligo adapters. After adapter ligation, PCR was carried out using DNA fragments with adapter molecules at both ends. PCR products were size selected (~625 bp) by 2 % agarose gel electrophoresis. Libraries were subjected to 50-bp-end multiplex sequencing on the Illumina HiSeq^™^ 2000 platform. After automatically removing adapter sequences and low-quality reads, high-quality paired-end reads were aligned to the NCBI human reference genome hg19 using the Short Oligonucleotide Analysis Package 2 alignment tool [15].

### Validation of the breakpoint regions by PCR and Sanger sequencing

Genomic DNA sequences flanking putative breakpoint regions were extracted from hg19 for further identification. For the amplification of putative junction site regions, validation primers were designed using primer software (Primer3, http://simgene.com/Primer3) with standard parameters. Primers and PCR conditions are available on request. Putative fragments were amplified by PCR using these primers, then PCR products were purified and sequenced on an ABI 3730*xl* DNA analyzer.

### Sequence analysis of junction fragments

The sequences of junction fragments were aligned to the human genome reference sequence (hg19) using Blast from NCBI. Analysis with the genomic context of the breakpoints was performed using the UCSC Genome Browser (http://genome.ucsc.edu/cgi-bin/hgGateway).

## Results

### Cytogenetics and cell culture

**Fig. 1:**
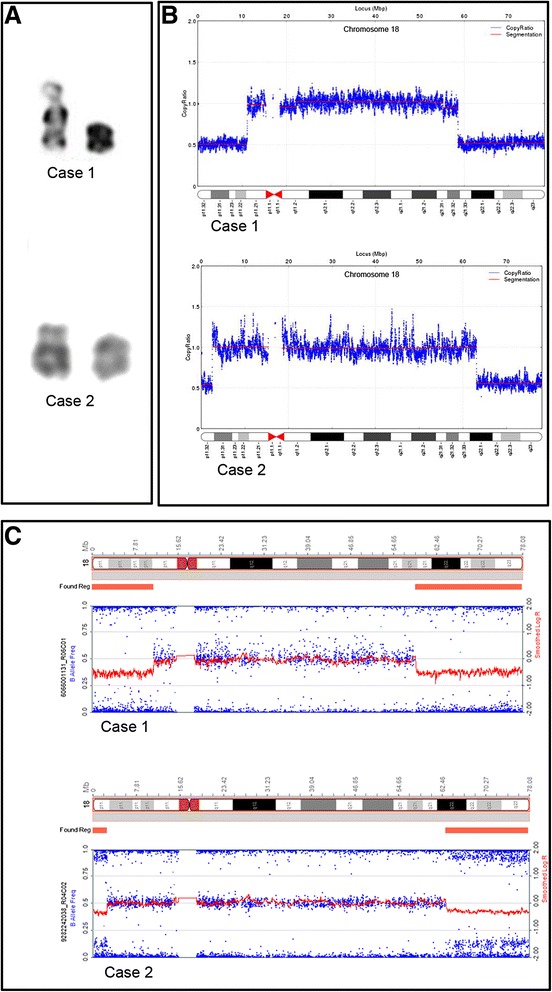
Deletion on both ends of the chromosome 18. **a** Karyotypes of chromosome 18 in both cases. The normal chromosome is shown on the left and the abnormal one on the right. **b** CNV detected by NGS. **c** CNV detected by the SNP array

Using conventional cell cultures and standard chromosomal preparations, G-banding analysis implied two suspected cases of r(18) (Fig. 1a). The karyotypes were initially designated 46,XX, r(18)[27]/45,XX,-18[5]? for case 1 and 46, XX, r(18)? for case 2.

### NGS and SNP array

Paired-end NGS analysis of both cases revealed genomic deletions. Case 1 was shown to have an 11 Mb deletion (chr18:111,935-11,175,737) within 18p11.32-p11.21 and a 19.39 Mb deletion (chr18: 58,568,271-77,958,754) within 18q21.32-q23. In case 2, a 2.4 Mb deletion (chr18: 138,005-2,541,233) was detected within 18p11.32 and a 14.9 Mb deletion (chr18: 63,108,020-78,013,427) within 18q22.1-q23 (Fig. 1b).

We performed SNP array to confirm these results. Correspondingly, an 18p11.32-p11.21 deletion (chr18: 12,842-11,176,068) and an 18q21.32-q23 deletion (chr18: 58,662,423-78,014,582) were reported in case 1. Case 2 was shown to have an 18p11.32 deletion (chr18: 12,842-2,548,128) and an 18q22.1-q23 deletion (chr18: 63,129,673-78,014,582) (Fig. 1c) (Additional file 1). These fingdings confirmed the presence of terminal deletions on both arms of chromosome 18 in both cases, which indicated the possibility of the formation of r(18) according to the previous reports [5].

### Breakpoint mapping

**Fig. 2:**
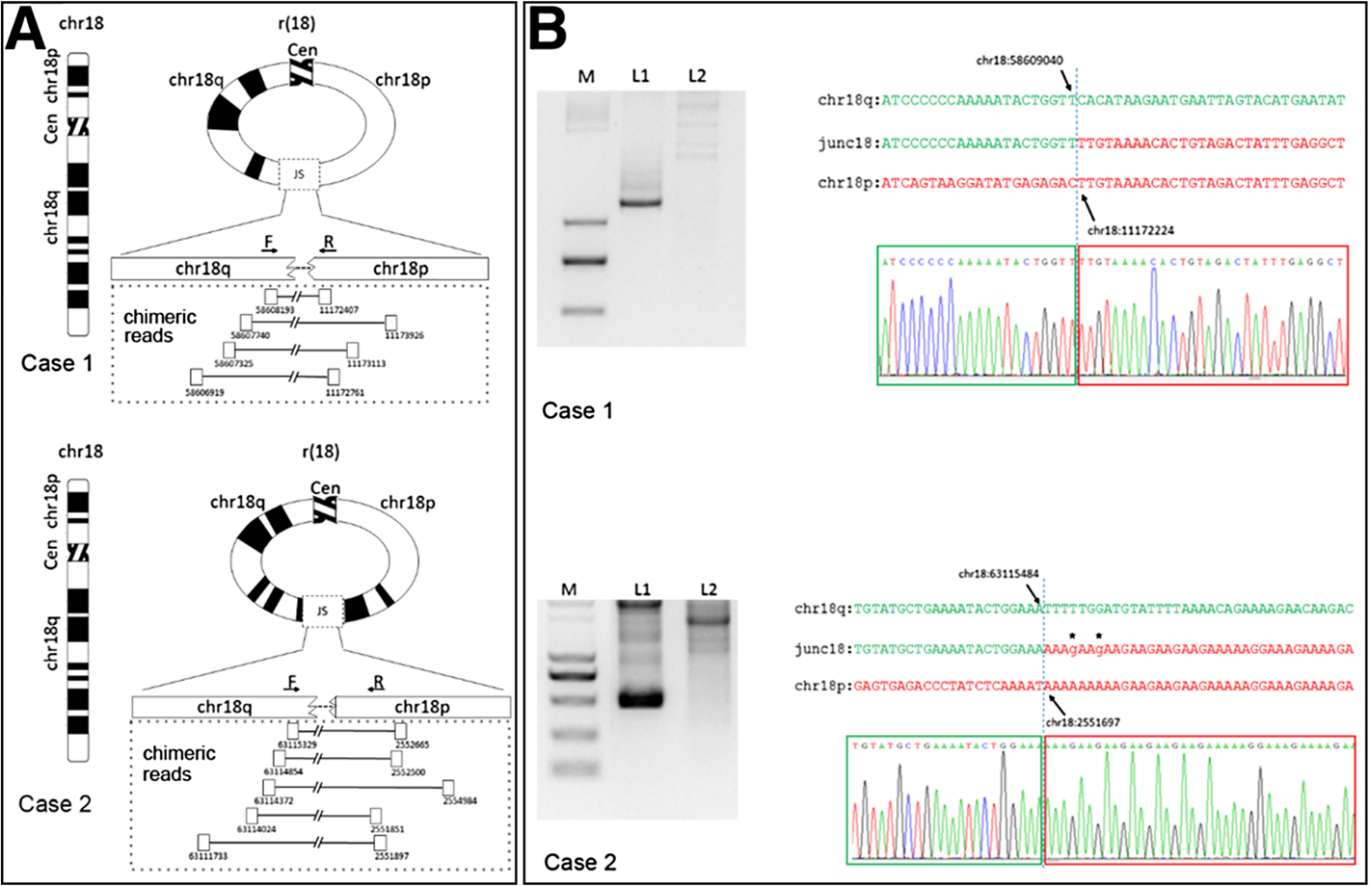
Characterization of r(18) and mapping of the breakpoints. **a** Schematic of chimeric mate-pair reads on chromosome 18 spanning the putative junction site (JS) in both cases. **b** Junction site sequences amplified by PCR (left, L1) and breakpoints (arrows) defined by Sanger sequencing (right). Genomic DNA from healthy individual was used as a negative control (left, L2). Two nucleotide variations on junction fragments in case 2 are marked in lower case and asterisked

Based on the NGS data, we analyzed chimeric mate-pair reads with both ends mapping to different genomic regions. We detected four and five chimeric mate-pairs spanning the putative junction sites of chromosome 18 in case 1 and case 2, respectively (Fig. 2a, Table 1). On the basis of their position, the breakpoints were estimated to be located 5' to chr18: 11172407 and 3' to chr18: 58608193 in case 1 and 5' to chr18: 2551851 and 3' to chr18: 63115329 in case 2.

**Table 1:** Information about chimeric mate-pair reads on chromosome 18 in both cases

Case 1					
chr	location	chr	location	reads direction	
chr18	11173926	chr18	58607740	+	-
chr18	11173113	chr18	58607325	+	-
chr18	11172407	chr18	58608193	+	-
chr18	11172761	chr18	58606919	+	-
Case 2					
chr	location	chr	location	reads direction	
chr18	2551851	chr18	63114024	+	-
chr18	2551897	chr18	63111733	+	-
chr18	2552500	chr18	63114854	+	-
chr18	2552665	chr18	63115329	+	-
chr18	2554984	chr18	63114372	+	-

To validate this result, we designed primers targeting the sequences flanking the putative junction sites. We successfully amplified the sequences spanning the junction site regions by PCR from patient genomic DNA but not control genomic DNA. Sanger sequencing identified the breakpoints in case 1 at position 11172224 and 58609040 on chromosome 18, and at position 2551697 and 63115484 on chromosome 18 in case 2. Two base pairs near the junction site not aligned to chromosome 18, and not known SNPs, were identified in case 2 (Fig. 2b). These results provide direct evidence for ring chromosome formation at the molecular level in both cases.

**Fig. 3:**
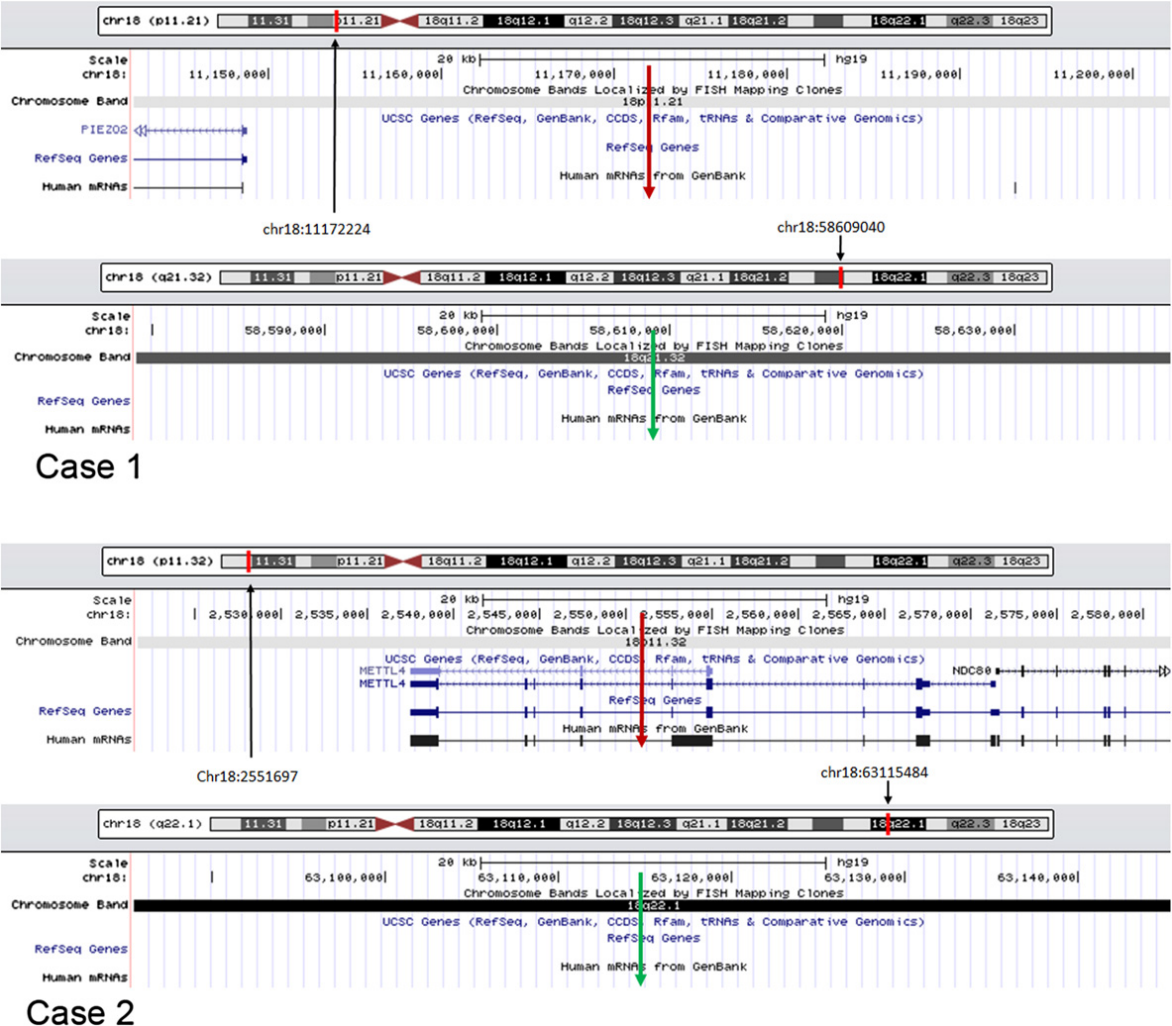
Chromosome breakpoints and disrupted genes. Breakpoints are indicated by red and green arrows. No genes were disrupted by any of the breakpoints in case 1 or by one of the breakpoints in case 2. The second breakpoint in case 2 on the long arm of chromosome 18 disrupted *METTL4*

We next analyzed the genomic location of the two breakpoints in both cases using the UCSC Genome Browser (https://genome.ucsc.edu/). In case 1, neither breakpoints disrupted any reference genes. In case 2, one breakpoint was located within the fifth intron of the methyltransferase like four gene (*METTL4*) , while the second did not disrupt any reference genes, indicating that no fusion gene was formed (Fig. 3). We also analyzed the interspersed repetitive elements which have been implicated in chromosome rearrangement [16]. Through repeat masker analysis in UCSC Genome Browser, we found that breakpoint 1 (chr18: 11172224) in case 1 was located within a long interspersed element (LINE) belonging to the L1 family, while breakpoint two (chr18: 58609040) was 188 bp away from a short interspersed element (SINE) belonging to the Alu family. In case 2, breakpoint one (chr18:2551697) was within a SINE belonging to the Alu family, and breakpoint 2 (chr18: 63115484) was 34 bp from a SINE belonging to the Alu family.

## Discussion

In the present study, we found two suspected cases of r(18) by GTG method (Fig. 1a), then used low-coverage whole-genome paired-end NGS to characterize the cases at a high resolution. We identified chromosome deletions on both arms, and the chromosome junctions at a resolution level of hundreds of base pairs. We also validated the chromosome deletions by SNP-array analysis, and used Sanger sequencing to characterize the r(18) breakpoints and confirm the chromosome junctions. Our findings show that this approach successfully detected all CNVs in the genome, and provided direct evidence of ring chromosome formation at a high level of resolution.

In the clinic, prenatal phenotypes of r(18) usually manifest as increased nuchal fold thickness, congenital heart disease, ventriculomegaly, cebocephaly, single umbilical artery, oligohydramnios, and holoprosencephaly [17]. Consistently, we observed similar prenatal phenotypes in the present two cases. Additionally, we found nuchal cystic hygromas in case 1. The symptoms of the infant in case two, including microcephaly, developmental retardation, orbital hypertelorism and ptosis of the upper eyelid, were also similar to other reports of r(18) [5].

The clinical features of r(18) usually correlate with the sizes and locations of the deleted genomic regions and junction sites. Ring chromosome identification in previous reports has mainly relied on the observation of ringed morphology by conventional G-banding analysis [3]. However, this method depends on clear banding, and has a low resolution. Although FISH can map breakpoints at a higher resolution and generate morphological evidence of ring formation, it is experimentally laborious and time-consuming, and cannot screen whole-genome rearrangements [4], which limits its clinical application. DNA arrays allow for a more accurate evaluation of whole-genome CNVs [5, 6], but cannot validate chromosomal rearrangements, including ring chromosomes. More accurate characterizations of r(18), such as achieved in the present study using NGS methods, will be helpful in the genetic counseling and management of prenatal cases.

Herein, a whole-genome low-coverage paired-end NGS based technology was applied to two cases with suspected r(18). We used a non-size selection (3 ~ 8 kb) mate-pair library with 40 M reads pairs, to give about 1.33× base coverage and around 66.7× physical coverage. We successfully detected chromosomal deletions and identified the breakpoint-spanning region by searching for chimeric mate-pair reads with both ends mapping to different chromosome arms. This approach was sufficiently powerful to detect deletions and breakpoints of r(18) with a low-coverage sequencing depth, and is suitable for the molecular characterization of ring chromosomes involving genome dose and structural changes.

Nonallelic homologous recombination (NAHR) [18] and inv-dup-del have been reported to be possible mechanism for the formation of chromosomal rearrangements [19]. However, our analysis of breakpoint regions did not identify any homologous sequences or fragments of inverted duplications flanking the breakpoints in either patient, suggesting that NAHR and inv-dup-del were not responsible for the formation of r(18) in our cases.

Recent studies have also suggested that repetitive sequences such as LINE and SINE elements may contribute to chromosome structural variations. Sobreira et al. [8] reported that five out of eight defined breakpoints were within repetitive sequences, while this was seen in nine out of ten breakpoints in a balanced translocation in a study by Schluth-Bolard [10]. Because very few ring chromosome breakpoints have been mapped and analyzed in detail before, the association between ring chromosomes and repetitive sequences is unknown. Repeat-masker analysis in the present study showed that two breakpoints were inside a LINE or SINE, and that the other two breakpoints were located near a LINE or SINE, indicating a possible association. However, further studies are necessary to confirm this.

Our study also explored the possibility of the generation of fusion genes generation as a result of the genomic rearrangement. No reference genes were disrupted by either of the two breakpoints in Case 1, or by one of the two breakpoints in case 2. However, the second breakpoint lied within the fifth intron of *METTL4* may lead to the loss of gene function. Nevertheless, it appeared that no fusion gene was formed, and that the clinical symptoms of two cases of r(18) were mainly caused by chromosomal deletions.

## Conclusions

We successfully characterized the chromosomal deletions and genomic junctions in two suspected cases of r(18) through a low-coverage whole-genome paired-end NGS analysis. This method appeared to be effective detecting genomic doses and structural changes in ring chromosomes with a high resolution. Our study also provides an insight into the genotype-phenotype correlations and the underlying mechanism of ring chromosome formation for future studies.

## Additional file

Additional file 1:
**Original SNParray data.** (XLSX 23 kb)

## Abbreviations

r(18): Ring chromosome 18
FISH: Fluorescence in situ hybridization
NGS: Next generation sequencing
CNVs: Copy number variations
SNP: Single nucleotide polymorphisms
aCGH: Array comparative genomic hybridization
VSD: Ventricular septal defects
GTG: G-band by trypsin using Giemsa
SINE: Short interspersed element
LINE: Long interspersed element
NAHR: Nonallelic homologous recombination
